# Alternative vs. conventional treatment given on-demand for gastroesophageal reflux disease: a randomised controlled trial

**DOI:** 10.1186/1472-6882-9-3

**Published:** 2009-02-24

**Authors:** Per G Farup, Mathis Heibert, Victor Høeg

**Affiliations:** 1 Department of Medicine, Innlandet Hospital Trust, Gjøvik, Norway; 2 Unit for Applied Clinical Research, Norwegian University of Science and Technology, Trondheim, Norway; 3 Department of Medicine, Nord-Trøndelag Hospital Trust, Namsos, Norway; 4 Department of Medicine, Innlandet Hospital Trust, Tynset, Norway

## Abstract

**Background:**

Alternative treatments are commonly used for various disorders and often taken on-demand. On-demand treatment of gastroesophageal reflux disease (GERD) with pharmaceutical products is an established, cost-effective strategy. Comparisons between alternative medicine and pharmaceutical products are rare. The aim of this trial was to compare on-demand treatment with a pectin-based, raft-forming, natural, anti-reflux agent (PRA) with that of esomeprazole 20 mg (Eso20) in patients with mild/moderate GERD.

**Methods:**

Patients with mild/moderate GERD were randomised to a six weeks' on-demand treatment with PRA or Eso20 in a pragmatic, open, multicentre trial. Overall satisfaction with treatment, satisfactory relief on a weekly basis, reflux symptoms, and treatment preferences were noted.

**Results:**

Seventy-seven patients were included in the analyses. Eso20 was significantly superior to PRA for proportion of overall satisfied patients (92% and 58% respectively; p = 0.001), reduction of symptoms (mean symptom scores at the end 5.9 and 8.0 respectively; p = 0.019), proportion of weeks of satisfactory relief (89% and 62% respectively; p = 0.008) and proportion preferring continuation with the same treatment (85% and 42% respectively; p < 0.001). Older patients were more satisfied than younger, and patients preferring on-demand treatment had lower symptom scores at inclusion than those preferring regular treatment.

**Conclusion:**

On-demand treatment with esomeprazole 20 mg was clearly superior to the pectin-based raft-forming agent. Most patients preferred on-demand treatment to regular treatment. Those preferring regular therapy had significantly more symptoms at inclusion.

**Trial registration:**

ClinicalTrials.gov: NCT00184522.

## Background

On-demand treatment of mild/moderate gastroesophageal reflux disease is an effective and cost-reducing strategy for long-term management [1-4]. The goal of reflux treatment is not necessarily complete absence of symptoms and normalisation of minor endothelial lesions, but satisfactory relief of symptoms, healing of major esophageal lesions and prevention of complications [5]. If an effective, fast-acting and sufficient long-lasting therapy is easily at hand, the patients seem to accept some intermittent complaints and on their own chose on-demand treatment [6-8]. There is an increasing use of complementary and alternative medicine (CAM) that, in contrast to drugs, is believed to be harmless. These products are often used on-demand.

On-demand treatment with esomeprazole has a well-documented effect in placebo-controlled trials [1,5] It is, however, probably not the ideal drug for on-demand treatment because the onset of action is delayed and maximal effect appears after several doses. CAM is preferred by many patients and deserves attention and comparisons with pharmaceutical products. Aflurax^® ^(Ferrosan AS) is a pectin-based, raft-forming natural agent approved for sale over the counter for mild/moderate reflux symptoms [9-12]. It has been marketed as an innocent, natural, pectin-based, locally acting, non-absorbable product with a rapid onset of effect, and is preferred by many patients, especially pregnant women.

On-demand treatment is an established administration schedule, but there are few comparisons between on-demand treatment with different drugs and little information about the characteristics of patients preferring this dosage schedule.

This open randomised controlled trial compared the symptomatic effect of on-demand treatment with a pectin-based, raft-forming, natural, anti-reflux agent with that of esomeprazole (Nexium^® ^AstraZeneca) 20 mg, and studied patient satisfaction with the products and the administration schedule.

## Methods

### Subjects

Nine outpatient clinics included consecutive patients above 18 years of age with mild/moderate heartburn/regurgitation as main symptom for more than 3 months and symptoms at least two days per week the last two weeks. Mild symptoms were defined as symptoms not interfering with daily activities, and moderate as symptoms interfering with daily activities but not interrupting or avoiding daily activities. Patients with mild dyspepsia or irritable bowel syndrome were included if heartburn/regurgitation clearly was the main complaint. A gastroscopy was performed and patients with non erosive reflux disease (NERD) and esophagitis Los Angeles grade A and B were included. Patients in need of continuous treatment as judged by the responsible physicians, were excluded, as were patients who had taken acid secretion inhibitors or antacids for five or more days the last two weeks, patients who clearly preferred continuous treatment, patients with other diseases that could influence the assessment, and those with anticipated poor compliance or significant drug or alcohol abuse. Pregnant or breast-feeding women and fertile women not practising a medically approved method of contraception were also excluded.

### Treatment regimens

The patients were randomized to treatment with one of the following regimens:

Esomeprazole (Nexium^®^, Astra-Zeneca) 20 mg tablets (Eso20) to be taken only when experiencing heartburn/regurgitation, upward limited to once daily.

A pectin-based, raft-forming, anti-reflux agent (Aflurax^®^, Ferrosan) (PRA) given as chewable tablets to be taken only when experiencing heartburn/regurgitation, upward limited to eight tablets per day. The product contains magnesium carbonate and potassium bicarbonate that react to form the raft. The raft acts as a physical barrier to reflux, or, if reflux occurs, the reflux consists of the raft and not acidic gastric content [12].

The drugs were taken to relieve symptoms, not to prevent symptoms, unlike intermittent treatment when drugs are taken regularly for short periods with symptoms.

### Study design

The study was a pragmatic, open, multi centre, block randomized (with variable block size, allocation ratio one, and stratified for centres and NERD versus esophagitis) clinical trial with a parallel group design. Randomization was computer based at the Unit for Applied Clinical Research, Faculty of Medicine, Norwegian University of Science and Technology who allocated the patients to one of the treatment groups after a phone call from the centre. The study duration was six weeks with visits at inclusion and at the end.

### Variables

At inclusion, all patients had socio-demographic information noted, a medical history taken, a physical examination and gastroscopy with description of esophagitis according to the Los Angeles classification performed, and haematological and biochemical tests taken when needed. Tests for *H. pylori *and life style modifications were not part of the study and were performed according to the doctors' discretion.

The patients filled in a reflux symptom questionnaire at inclusion and at the end. Five questions regarding reflux symptoms from the Gastrointestinal Symptom Rating Scale (GSRS) were scored on a seven point Likert scale from 0 – 6, giving a symptom score with range 0 – 30 [13].

The patients answered three questions on a diary card at the end of each treatment week: 1: Have you had heartburn/regurgitation the last week (Yes/No)? 2: Have you taken study drugs the last week (Yes/No)? 3: Did the intake of drugs result in satisfactory relief of symptoms the last week (Yes/No)? The proportion of weeks of satisfactory relief of drug treatment was calculated.

At the last visit, unused medication was returned and counted and the patients were asked for overall satisfaction with the treatment (yes/no), preference for further drug treatment (continue with the same drug or switch to another drug), preference for on-demand versus continuous treatment, and side effects. Spontaneously reported side effects were recorded and the following foreseeable inconveniences/side effects were asked for (yes/no): Was the time until effect of the tablets too long? Did the symptoms recur too early? Was the number of tablets too high? Did you have any inconveniences with the intake of the drug?

### Statistics

The analyses were performed with t-test (for variables with normal distribution), Mann-Whitney U-test, Fisher's exact test, Chi-square test with linear-by-linear association when appropriate and logistic regression analyses by means of the statistical package SPSS^® ^v.13 with exact tests when available in the package. Two-tailed significance tests were used and p-values < 0.05 were regarded as statistically significant.

The main analysis was a modified intention to treat (ITT) analysis. Missing data were replaced by carrying the last observation forward or imputation of overall mean if no previous observations were available. Patients with complete data, i.e. at least four weeks with correctly filled in diary cards, were included in the per protocol analyses.

The study was designed to show non-inferiority of PRA to Eso20. Eso20 was presumed to give overall satisfaction in 80% of patients, and non-inferiority was defined as an effect of PRA that was at most 10% inferior to Eso20. The number of patients necessary to show this equivalence is 200 in each group (α = 0.05, β = 0.20, one-sided test). Because an impression arose that PRA was less effective than Eso20, an interim analysis was performed and the trial was terminated prematurely. The results are given as mean and SD if not otherwise indicated.

### Ethics

The trial was conducted according to the Declaration of Helsinki and approved by The Regional Committee for Medical Research Ethics in Trondheim, Norwegian Social Science Data Services (NSD) and Norwegian Medicines Agency (SLV); Norway. All patients got oral and written information about the trial and gave written informed consent to participate before inclusion in the trial.

## Results

**Figure 1:**
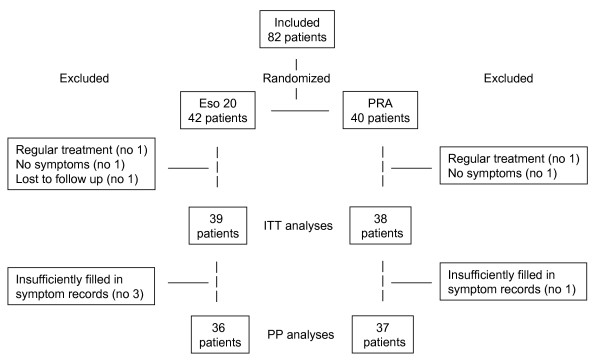
**The figure shows the flow of patients through the trial**. (Eso20 = Esomeprazole 20; PRA = Pectin-based, raft-forming agent; ITT = Intention to treat; PP = Per protocol).

**Figure 2:**
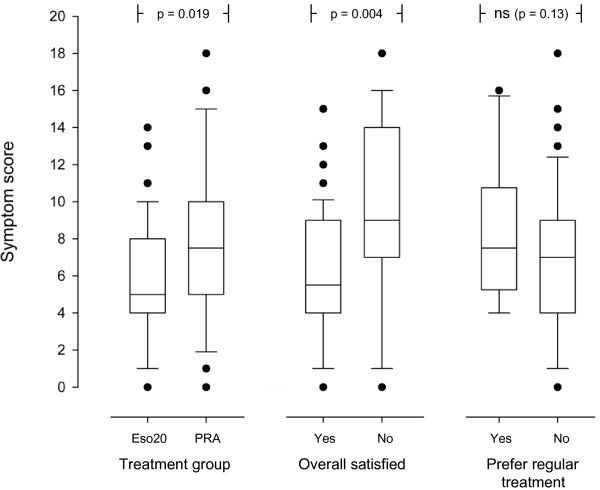
**The box plot shows the reflux symptom scores (10^th^, 25^th^, 50^th^, 75^th ^and 90^th ^percentiles and outlying values) at the end of the study related to treatment group, overall satisfaction and preference for regular treatment**. (Eso20 = Esomeprazole 20 mg; PRA = Pectin-based, raft-forming agent).

Eighty-two patients were included, 77 were available for the ITT analyses and 73 for the PP analyses. Figure 1 shows the flow of patients through the trial. Table 1 gives the patients' characteristics. There were no significant differences between the two treatment groups at inclusion. Fifty-eight patients (75%) were overall satisfied with the treatment. Table 2 gives the results of the comparisons between the two treatment groups. Eso20 was significantly superior to PRA for overall satisfaction with treatment, number of weeks with satisfactory relief of symptoms, reduction of symptoms and preference for continuation with the same treatment. The comparisons between patients with and without overall satisfaction with the treatment are shown in table 3. Overall satisfied patients were significantly older, smoked less, had lower symptom score at the end and more weeks with satisfactory relief of symptoms. Symptom score at inclusion and at the end were not correlated with age (r = 0.048; p = 0.68 and r = -0.17; p = 0.13 respectively). Symptom score at inclusion in patients who at the end preferred regular and on-demand treatment were 13.8 (4.3) and 10.9 (3.8) respectively (p = 0.017). There were no other differences between the groups preferring regular and on-demand treatment (data not shown). Figure 2 shows symptom score at the end in different groups of patients. Independent predictors (logistic regression analyses) for overall satisfaction were: Treatment group (Eso20) (OR = 8.53; 95% CI: 1.81–40.13; p = 0.007) and older age (OR = 1.066; 95% CI: 1.01–1.12; p = 0.02) with a trend for a relation to low symptom score at the end (OR = 1.167; 95% CI: 0.988–1.377; p = 0.068) and non smoking (OR = 2.247; 95% CI: 0.941–28.905; p = 0.069). The per protocol analyses showed corresponding results (data not shown).

**Table 1:** Characteristics of the subjects in the two treatment groups.

**Patients' characteristics**	**Eso20**	**PRA**
Number of subjects	39	38
Male (no)	22 (56%)	21 (55%)
Age in years (mean)	47.8 (14.5)	46.2 (14.8)
BMI (mean)	26.2 (3.1)	25.7 (3.0)
Cups of coffee per day (mean)	3.0 (2.2)	3.3 (2.6)
Smokers (daily/past/never) (no)	9/12/18	10/16/12
Duration of reflux symptoms in years (mean)	8.0 (7.6)	8.0 (8.5)
Symptom score at inclusion (mean)	11.3 (3.9)	11.3 (4.1)
Degree of esophagitis (no)		
- NERD	13 (33%)	15 (40%)
- Los Angeles Grade A	20 (51%)	17 (45%)
- Los Angeles Grade B	6 (15%)	6 (16%)
Hiatal hernia (no)	26 (67%)	24 (63%)

The groups were well balanced. The results are given as number of patients (proportion in brackets) or mean (standard deviation in brackets).

Eso20: Esomeprazole 20 mg.

PRA: Pectin-based Raft-forming Agent.

BMI: Body mass index.

NERD: Non erosive reflux disease.

**Table 2:** Effect and side effects in the two groups.

**Effect and side effects**	**Eso20**	**PRA**	**Statistics**
Overall satisfaction with the treatment (no)	36 (92%)	22 (58%)	p = 0.001
Weeks of satisfactory relief (mean proportion)	89% (26)	62% (44)	p = 0.008
Symptom score at the end of treatment (mean)	5.9 (3.4)	8.0 (4.4)	p = 0.019
Preference for same drug after the trial (no)	33 (85%)	16 (42%)	P < 0.001
Preference for regular treatment (no)	6 (15%)	6 (16%)	ns (p = 1.00)
Diarrhoea (no)	1 (3%)	6 (16%)	ns (p = 0.056)
Too many tablets (no)	2 (6%)	11 (29%)	p = 0.006
Delayed onset of effect (no)	10 (26%)	4 (11%)	ns (p = 0.14)
Rapid relapse of symptoms (no)	8 (21%)	18 (47%)	p = 0.017

The results are given as number of patients (proportion in brackets) or mean (standard deviation in brackets).

Eso20: Esomeprazole 20 mg.

PRA: Pectin-based Raft-forming Agent.

**Table 3:** The characteristics of subjects with and without overall satisfaction with the treatment.

**Patients' characteristics**	**Overall satisfied**		**Statistics**
		
	**Yes**	**No**	
Number of subjects	58	19	
Male (no)	32 (55%)	11 (58%)	ns (p = 1.00)
Age in years (mean)	49.6 (14.8)	39.2 (10.6)	p = 0.002
BMI (mean)	25.8 (2.6)	26.3 (3.8)	ns (p = 0.51)
Cups of coffee per day (mean)	3.0 (2.2)	3.4 (3.0)	ns (p = 0.72)
Smoking (daily/past/never) (no)	13/17/28	6/11/2	p = 0.029
Duration of reflux symptoms in years (mean)	7.7 (7.7)	9.0 (8.9)	ns (p = 0.55)
Esophagitis (NERD/LAgrA/LAgrB) (no)	10/28/20	2/9/8	ns (p = 0.46)
Hiatal hernia (no)	21 (36%)	6 (32%)	ns (p = 0.78)
Symptom score at inclusion (mean)	11.2 (3.6)	11.9 (5.0)	ns (p = 0.49)
Symptom score at the end of treatment (mean)	6.1 (3.4)	9.5 (4.8)	p = 0.004
Weeks of satisfactory relief (mean proportion)	94.6% (13.6)	18.7% (32.0)	p < 0.001
Prefer same drug after the trial (no)	48 (83%)	1 (5%)	p < 0.001
Prefer regular treatment (no)	7 (12%)	5 (26%)	ns (p = 0.15)
Rapid relapse of symptoms (no)	15 (26%)	11 (58%)	p = 0.023
Delayed onset of effect (no)	9 (16%)	5 (26%)	ns (p = 0.32)
Too many tablets (no)	6 (10%)	7 (37%)	p = 0.013
Diarrhoea (no)	6 (10%)	1 (5%)	ns (p = 0.67)

The results are given as number of patients (proportion in brackets) or mean (standard deviation in brackets).

BMI: Body mass index.

NERD: Non erosive reflux disease.

LAgrA: Los Angeles grade A.

LAgrB: Los Angeles grade B

The mean daily use of Eso20 was 0.59 tablets (95% CI 0.49 – 0.68). Misunderstandings hampered reliable calculation of the intake of PRA.

## Discussion

Esomeprazole was clearly superior to PRA. Patients on esomeprazole reported a higher prevalence of overall satisfaction with the treatment, had more weeks of satisfactory symptom relief, a lower symptom score the last two weeks and preferred more often to continue the same treatment. This superiority of established pharmacological treatment over CAM explains the infrequent use of alternative medicine for reflux disease compared to the use for other disorders [14]. However, it was not an ideal treatment. Delayed onset of effect and rapid relapse of symptoms were noted by one out of four and five patients respectively. Satisfactory relief of symptoms failed every 10^th ^week, 15% asked for another drug and 15% preferred regular treatment.

PRA was less effective, but more than half of the patients were satisfied with the treatment and nearly half of them wanted to continue the treatment. The main advantage of PRA was a trend toward a faster acting effect. Disadvantages were rapid relapse of symptoms, intake of too many tablets, and some diarrhoea.

The placebo response in mild/moderate reflux disease is substantial. This study allows no comparisons with placebo, but PRA is probably more effective than placebo. Other raft-forming agents have shown an immediate reduction in gastro-oesophaeal reflux and increase in oesophageal pH, which is superior to that of omeprazole for 4 hours [15]. PRA reduces reflux of both food and acid. The reduced reflux of food and not only acid shows the rafting properties of PRA, which seem to be at least as good as those of comparable anti-reflux agents [12]. Two placebo-controlled clinical trials have proven a symptomatic effect of PRA above placebo [9,11]. The placebo response rates vary a lot in clinical studies but are in most studies inferior to that of PRA in this study [1,5]. Therefore, PRA seems to be an alternative for a minority of patients with mild/moderate reflux disease who prefer natural, locally acting, non-absorbable agents rather than ordinary pharmaceutical products.

Evidence Based Medicine has to become involved into CAM as long as our patients prefer, use and report effects of such products. We need knowledge, but unlike this trial, CAM-producers seldom support high quality research. This study indicates that alternative products could have a place in the therapeutic armamentarium.

Overall satisfaction was the main outcome in the trial. This outcome depends on factors like symptomatic effect, drug administration schedule (e.g. on-demand versus continuous treatment, or dosing once versus several times daily) number of tablets, drug formulation (e.g. small or large tablets, chewable tablets, or granules), taste, preference for natural or pharmaceutical products, age, disease under study, expectation and experience from previous treatment regimens etc. This study does not allow conclusions about all factors related to overall satisfaction. The most important predictor for overall satisfaction was treatment with esomeprazole. This is likely due to the efficient relief of symptoms, but not only. The convenient dosage schedule with one tablet a day, the rather long-lasting effect, the good tolerance and perhaps previous experience with acid secretion inhibitors might have contributed to overall satisfaction with esomeprazole. Symptom score last two weeks showed an insignificant trend toward prediction of overall satisfaction after correction for treatment group and indicates that symptom relief is not decisive for satisfaction.

Overall satisfaction increased with age. Since symptoms did not correlate with age, other factors such as habituation to and tolerance for minor complaints and gratitude for some symptom relief might increase with age and explain the increased satisfaction in the elderly.

Patient preference for continuing on-demand treatment was impressive and in accordance with other studies [6,7]. On-demand treatment will necessarily result in more symptoms than continuous therapy does, but despite of more symptoms patients are nearly as satisfied with on-demand treatment as continuous treatment [8]. Twelve patients (16%) wanted to switch to regular treatment after the trial. The perfect drug for on-demand treatment has an immediate, long-lasting and sufficient effect [16], which is not fulfilled by any of the actual drugs. A high symptom score at inclusion was the only predictor for continuous treatment, not the degree of symptoms at the end. Even with an ideal drug, it is likely that on-demand treatment is unacceptable for some patients since it implies relapses. Continuous treatment is probably preferable especially if symptoms during relapses are severe. It is therefore comprehensible that patients with a high symptom score at inclusion also perceive severe relapses and prefer regular dosing.

On-demand treatment is not a precise definition. The term includes treatment of actual symptoms as in this trial, treatment to prevent foreseeable symptoms, intermittent therapy defined as regular intake of drugs for a short period when needed, and threshold therapy in which the patients adjust the medication down to a dose and frequency that still maintains adequate control of symptoms [5]. This trial, in which patients took one tablet when needed to relieve symptoms, gives no information about other on-demand dosage schedules. The intake of approximately one tablet of Eso 20 every other day indicates that the patients have taken the drugs as prescribed.

## Conclusion

Overall satisfaction was significantly higher in patients given esomeprazole 20 mg than in those given the pectin-based raft-forming natural agent, and increased with age. Most patients preferred on-demand treatment. The minority who preferred continuous therapy had significantly higher symptom scores at inclusion.

## Competing interests

Ferrosan AS, Norway/Denmark partially funded the study and provided one of the compounds employed in the trial.

## Authors' contributions

PGF was the main contributor to the concept and design of the study, took part in acquisition of data, was responsible for the analyses and interpretation of the data, and has drafted and revised the manuscript.

MH participated in the design of the study, acquired a substantial part of the data, and participated actively in the analyses and interpretation of the data and drafting of the manuscript.

VH acquired a substantial part of the data and participated in drafting of the manuscript.

All authors approved the published version.

## Pre-publication history

The pre-publication history for this paper can be accessed here:


